# Modulation of Innate Immune Signalling by Lipid-Mediated MAVS Transmembrane Domain Oligomerization

**DOI:** 10.1371/journal.pone.0136883

**Published:** 2015-08-28

**Authors:** Luis Nobre, Daniel Wise, David Ron, Romain Volmer

**Affiliations:** 1 Cambridge Institute for Medical Research, University of Cambridge, Cambridge, United Kingdom; 2 Wellcome Trust MRC Institute of Metabolic Science, Cambridge, United Kingdom; 3 NIHR Cambridge Biomedical Research Centre, Cambridge, United Kingdom; 4 Université de Toulouse, INP, ENVT, UMR1225, IHAP, F-31076 Toulouse, France; 5 INRA, UMR1225, IHAP, F-31076 Toulouse, France; National Institute of Allergy and Infectious Diseases—Rocky Mountain Laboratories, UNITED STATES

## Abstract

RIG-I-like receptors detect viral RNA in infected cells and promote oligomerization of the outer mitochondrial membrane protein MAVS to induce innate immunity to viral infection through type I interferon production. Mitochondrial reactive oxygen species (mROS) have been shown to enhance anti-viral MAVS signalling, but the mechanisms have remained obscure. Using a biochemical oligomerization-reporter fused to the transmembrane domain of MAVS, we found that mROS inducers promoted lipid-dependent MAVS transmembrane domain oligomerization in the plane of the outer mitochondrial membrane. These events were mirrored by Sendai virus infection, which similarly induced lipid peroxidation and promoted lipid-dependent MAVS transmembrane domain oligomerization. Our observations point to a role for mROS-induced changes in lipid bilayer properties in modulating antiviral innate signalling by favouring the oligomerization of MAVS transmembrane domain in the outer-mitochondrial membrane.

## Introduction

Detection of viruses by pathogen associated molecular pattern (PAMP) receptors leads to the secretion of type I interferons (IFN), which bind to IFN receptors and stimulate the expression of genes that inhibit virus replication and spread. Detection of viral nucleic acids by Toll-like receptors and RIG-I-like receptors (RLRs) initiates this antiviral innate immune response [1]. RLRs comprise RIG-I, which binds to viral 5’-triphosphate RNA, MDA5, which detects viral double stranded RNA, and LGP2, which regulates RIG-I and MDA5 signalling. Binding of viral RNA to RIG-I and MDA5 triggers a conformational change in these proteins that exposes their Caspase Activation and Recruitment Domains (CARD). RIG-I and MDA5 subsequently bind to and activate the mitochondrial antiviral signalling protein (MAVS, also known as IPS-1, VISA, and CARDIF), presumably via the interaction of the RLR CARD domain with MAVS CARD domain [2]. MAVS undergoes RLR-induced formation of higher order structures ranging from dimers to prion-like aggregates that lead to the recruitment and activation of signalling proteins culminating in the activation of the transcription factors IRF-3 and NF-κB and transcription of IFNs [3].

MAVS is a tail-anchored membrane protein inserted in the outer mitochondrial membrane (OMM), and is also found in the peroxisomal membrane and the mitochondrial associated membrane of the endoplasmic reticulum [4,5]. The localization of MAVS seems to contribute to the regulation of downstream signalling, as peroxisomal MAVS has been implicated in the early type III IFN-dependent antiviral response, while mitochondrial MAVS was shown to mediate a longer lasting type I IFN dependent antiviral response [4,6]. These observations suggest that the specific protein or lipid composition of organelles or specific organelle-associated functions could modulate MAVS function. The mitochondrial protein mitofusin 1 (MFN1) has for example been shown to interact with MAVS and potentiate antiviral innate immune signalling [7,8]. Further evidence suggests that MAVS signalling is promoted by mitochondrial fusion and inhibited by mitochondrial fission, highlighting an enigmatic link between mitochondrial dynamics and MAVS signalling [7,9]. Mitochondrial membrane potential also modulates MAVS signalling by an unknown mechanism [10].

Because of their link to inflammation [11,12], mitochondrial reactive oxygen species (mROS) have emerged as particularly interesting members of this growing list of mitochondrial signals that modulate MAVS signalling [13,14]. Mitochondria are an important source of reactive oxygen species (ROS), which can be considered as by-products formed during the transfer of electron through the respiratory chain [15]. ROS oxidize proteins, nucleic acids and lipids, thereby impairing or modifying the function of these targets [16]. ROS are not merely destructive chemical agents, they have also important physiological roles as signalling molecules [17]. ROS have been shown to modulate innate immune signalling by activating p38 MAPK and promoting TRAF3-6 glutathionylation [18,19]. How mROS promote MAVS-mediated type I IFN production remains however unknown.

ROS are extremely reactive towards double bonds of unsaturated fatty acids causing the addition of a peroxyl radical in the acyl chain and displacing the double bond; a process called lipid peroxidation [20]. The incorporation of a peroxyl radical into the acyl chain dramatically changes its chemical nature, as the peroxyl radical is hydrophilic, while the acyl chain is hydrophobic. The presence of oxidized acyl chains destabilizes the hydrophobic core of the lipid bilayer, forcing the phospholipids to adopt a new conformation. As the conformation of the phospholipids affects the biophysical properties of the membrane, lipid peroxidation can affect membrane fluidity, membrane thickness and acyl chain flexibility [21].

The biophysical properties of membranes significantly influence the behaviour of transmembrane (TM) proteins within the lipid bilayer. Indeed, changes to the lipid bilayer biophysical properties have been shown to affect the oligomeric state of single pass TM proteins, independently of any ligand [22–24]. Oligomerization is both necessary and sufficient for MAVS activation, suggesting that factors influencing its propensity to oligomerize could modulate MAVS signalling [25,26]. These considerations prompted us to test the hypothesis that mROS-induced changes in the biophysical properties of the outer mitochondrial membrane potentiate antiviral innate immune signalling by promoting MAVS TM domain oligomerization through a simple biophysical principle.

## Materials and Methods

### Generation of Transgene Expressing Cells

A cDNA encoding a N-terminally FLAG-tagged mouse PERK kinase domain (amino acids 538–1113, numbering based on *Mus musculus* PERK–Uniprot Q9Z2B5) (PERK-KD) fused to the C-terminal containing transmembrane domain of human MAVS (amino acids 509–540, numbering based on Uniprot Q7Z434) (MAVS-TM) was synthesized by Genscript and cloned into the pCDNA5_FRT_TO plasmid (Invitrogen) to generate the FLAG_PERK-KD_MAVS-TM expression plasmid. A cDNA encoding the C-terminal containing transmembrane domain of the outer mitochondrial outer membrane protein 25 (OMP25-TM) (amino acids 109–145, numbering based on Uniprot P57105) was synthesized by Genscript and cloned into pCDNA5_FRT_TO_FLAG_PERK-KD_MAVS-TM to exchange the TM domain of MAVS with the TM domain of OMP25 and to generate FLAG_PERK-KD_OMP25-TM expression plasmid. These expression plasmids were co-transfected with the pOG44 plasmid (Invitrogen) into Flp-In T-Rex-293 cells (Invitrogen) to generate isogenic cells expressing the transgene under a doxycycline inducible promoter. Stable clones were selected by blasticidin selection. The cytosolic Fv2E-PERK construct and the generation of Fv2E-PERK expressing cells was previously described [27].

### Cell Treatments and Viral Infections

Cells were treated with rotenone, antimycin A, hydrogen peroxide at the doses and for the time indicated in the figure legends. Cells were treated with poly(I:C) complexed to the transfection reagent Lyovec (poly(I:C)/Lyovec, Invivogen) as described in the figure legends. Cells were infected with 100HA/ml of Sendai virus strain Cantell, obtained from Charles River laboratories. Infection was started in 10cm dishes with inoculation for 1h with virus diluted in 2ml DMEM, followed by removal of the inoculum and addition of DMEM medium containing 10% serum and antibiotics. Transgene expression was always induced by treatment with 100μM doxycycline for a period of 90min ending at the moment of cell harvest.

### Immunoprecipitation and Immunoblotting

Cells were lysed as previously described [28]. Anti-Flag affinity gel (Sigma) was used to immunoprecipitate Flag-tagged proteins followed by immunoblotting with mouse monoclonal antibody Flag M2 (Sigma). In vitro dephosphorylation was performed on immunoprecipitation beads with purified bacterially-expressed lambda phosphatase.

### Quantitative Reverse Transcription PCR (q-RT-PCR)

RNA was collected with a RNeasy Mini kit (Qiagen). RNA for human CXCL10, IFN-β and β-actin was analysed with premade Taqman Gene Expression Assay primers and probes (Applied Biosystems) and an RNA-to-CT 1-step kit (Applied Biosystems).

### ROS Detection by Flow Cytometry

Cells were treated as indicated in the figure legends and stained 30min before harvest with 2.5μM MitoSOX Red superoxide indicator (Invitrogen) at 37°C in a 5% CO_2_ incubator. Cells were then washed with phosphate buffered saline, trypsinized and processed for flow cytometry analysis.

### Confocal Microscopy

Cells were stained with the mitochondrial specific marker MitoTracker Red CMXRos (Invitrogen) and then fixed and processed for immunofluorescence as previously described [29]. Cells were stained with the mouse monoclonal antibody Flag M2 (Sigma) and secondary anti-mouse Dye Light 488. Images were taken on a Zeiss Meta 510 confocal microscope using sequential acquisition.

### Measurement of Lipid Peroxidation

Lipid peroxidation was measured with the Thiobarbituric Acid Reactive Substances (TBARS) assay (Cayman) according to the manufacturer’s protocol.

### Statistical Analysis

Statistical significance for poly(I:C) and rotenone treatment effects was determined by one-way analysis of variance with post-hoc Bonferroni test. Statistical significance for Sendai virus time-dependent effects was determined by one-way analysis of variance with post-hoc Dunnett’s test defining time 0h as the reference control group. Statistical significance for comparisons between treated and non-treated samples in the TBARS assay was determined with Mann-Whitney test.

## Results

**Fig 1:**
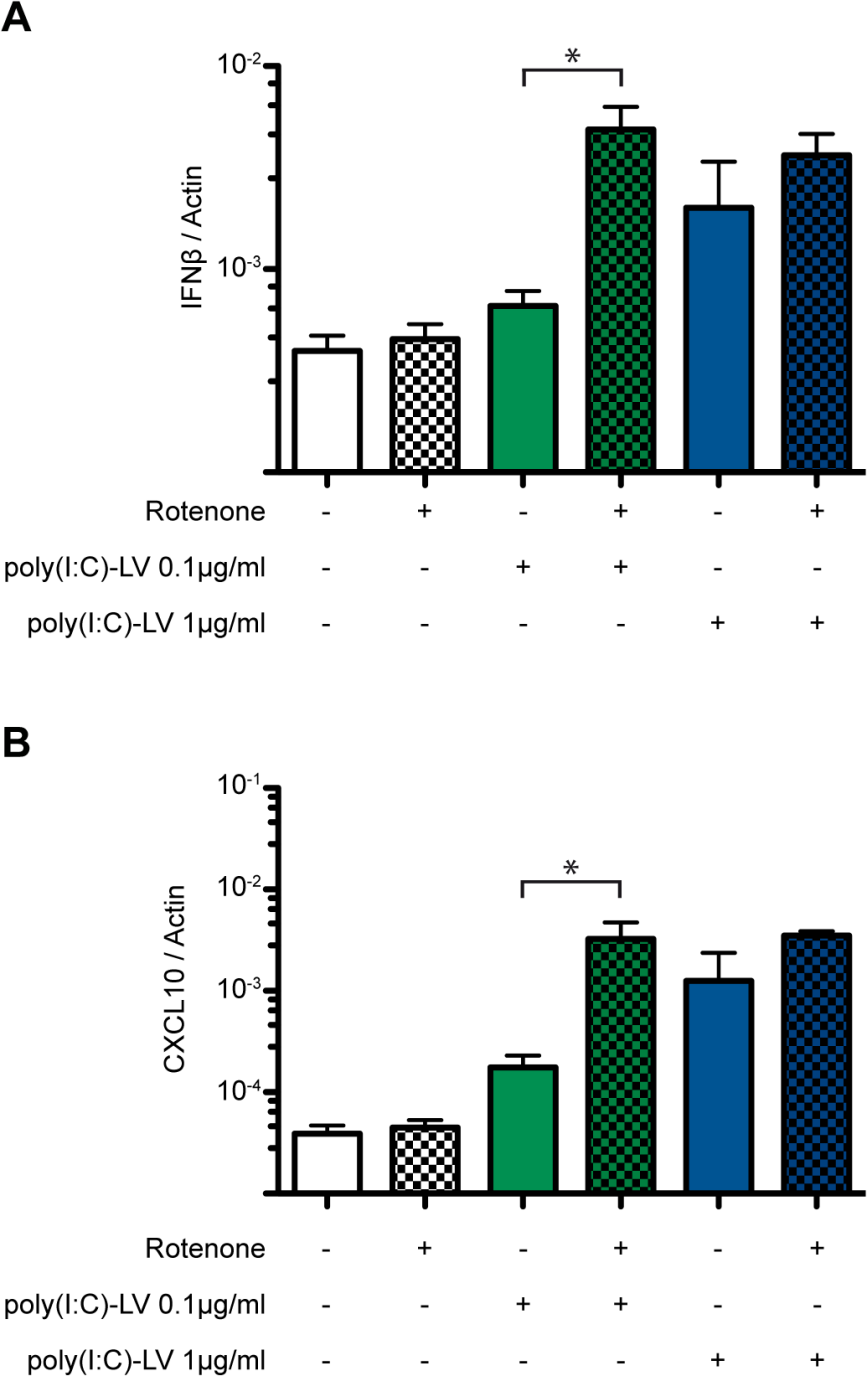
Rotenone potentiates RLR signalling. HEK 293T cells were treated with 1 μM rotenone alone or in combination with 0.1 μg/ml or 1 μg/ml of the intracellularly delivered RLR ligand poly(I:C)/Lyovec (poly(I:C)-LV) for 16h. **(A)** IFNβ mRNA levels and **(B)** CXCL10 mRNA levels were quantified by qRT-PCR and normalized to the level of β actin mRNA. Shown are the mean ± standard error of the mean (sem) of three independent experiments. *, p ≤ 0.05

To explore the consequences of increased mROS production on type I IFN responses, we treated the human HEK 293T cell line with rotenone [30]. Inhibition of mitochondrial respiratory chain complex I by rotenone enhanced mROS production, as reflected by increased MitoSOX staining (S1 Fig). When used alone, neither rotenone (at 1μM for 16h), nor the RLR ligand poly(I:C)-LyoVec (poly(I:C)-LV) (at 0.1μg/ml) led to increased IFN-β transcription (Fig 1A). By contrast, treatment of cells with both rotenone and 0.1μg/ml poly(I:C)-LV led to a five fold increase in IFN-β transcription (compared to cells treated with 0.1μg/ml poly(I:C)-LV alone), reaching IFN-β transcript levels similar to those observed in cells treated with 10 fold higher concentration of poly(I:C)-LV (1μg/ml) alone. Rotenone did not measurably increase IFN-β mRNA levels beyond those observed in cells treated with 1μg/ml poly(I:C)-LV, suggesting that IFNβ transcription had already reached a maximum. Similar upregulation of the chemokine CXCL10 transcripts were found in cells co-treated with rotenone and 0.1μg/ml poly(I:C)-LV (Fig 1B). CXCL10 is upregulated directly by PAMP receptor signalling and IRF3 mediated transcriptional activation or by type I IFN signalling [31]. These results indicate that rotenone potentiates RLR signalling only when submaximal concentrations of RLR ligands are present, leading to increased transcription of type I IFN.

**Fig 2:**
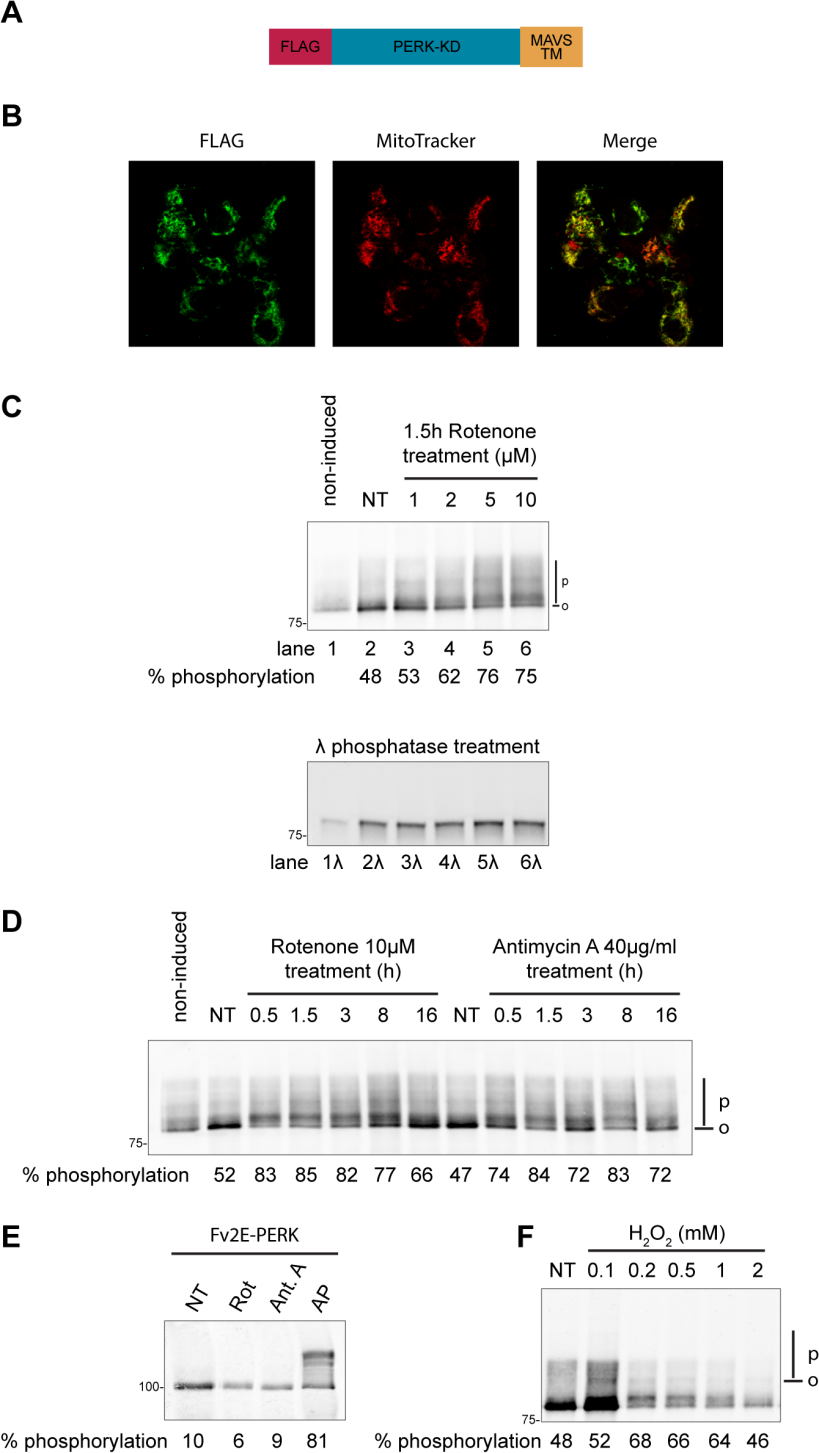
Increased MAVS-TM domain oligomerization in cells with elevated reactive oxygen species. **(A)** Schema of Flag_PERK-KD_MAVS-TM, a N-terminally Flag-tagged fusion protein between the kinase domain of PERK and the TM domain of MAVS. The construct encoding this protein was introduced into Flp-In T-Rex-293 cells by recombination **(B)** Intracellular localization of Flag_PERK-KD_MAVS-TM following doxycycline induction in Flp-In T-Rex-293 cells. Cells were stained for the Flag tag and the mitochondrial specific dye MitoTracker. Images were acquired sequentially on a confocal microscope. The merge panel shows an overlap of Flag and Mitotracker signals. **(C)** Immunoblot of Flag_PERK-KD_MAVS-TM immunopurified from Flp-In T-Rex-293 cells. Cells were either left untreated (non-induced) or exposed to doxycycline to induce the expression of Flag_PERK-KD_MAVS-TM. In addition, cells were treated for 90min with the indicated doses of rotenone. The position of hypophosphorylated (0) and phosphorylated (P) Flag_PERK-KD_MAVS-TM is indicated in the top panel. The bottom panel is an immunoblot of the same samples treated in vitro with λ-Phosphatase. **(D)** Immunoblot of Flag_PERK-KD_MAVS-TM immunopurified from Flp-In T-Rex-293 cells treated with 10 μM rotenone or 40 μg/ml antimycin A for the indicated time. **(E)** Immunoblot of Fv2E-PERK, immunopurified from cells treated with 10 μM rotenone (Rot) or 40 μg/ml antimycin A (Ant A), or with the Fv2E-PERK dimerizing ligand AP20187 (AP) at 10 nM for 90 min. **(F)** Immunoblot of Flag_PERK-KD_MAVS-TM immunopurified from Flp-In T-Rex-293 cells exposed for 2 hours to the indicated concentrations of hydrogen peroxide. The percentage of PERK-KD phosphorylation is indicated under each sample.

To test if increased mROS production could promote MAVS-TM domain oligomerization, we engineered a chimeric protein formed by the N-terminally FLAG-tagged catalytic domain of the endoplasmic reticulum stress kinase PERK (PERK-KD) fused to the transmembrane domain (TM) of MAVS (Flag_PERK-KD_MAVS-TM, Fig 2A). Oligomerization of the kinase domain of the endoplasmic reticulum stress transducer PERK promotes autophosphorylation, which is readily detectable on a SDS-PAGE gel by the appearance of slower migrating bands corresponding to phosphorylated forms of the protein [32]. When PERK-KD is fused to a TM domain, changes in the lipid membrane composition have been shown to cause lipid-dependent phosphorylation of PERK-KD that is driven by TM-domain oligomerization [24]. We therefore anticipated that PERK-KD fused to MAVS-TM domain would serve as a sensitive reporter on MAVS-TM domain oligomerization should it occur in response to changes in the outer mitochondrial membrane biophysical properties.

Single copies of Flag_PERK-KD_MAVS-TM were introduced by recombination into a specific genomic location of the Flp-In T-Rex-293 cell line. Flp-In T-Rex-293 cells also contain a Tet-ON inducible system to control the expression of the transgene. Following doxycycline induction, Flag_PERK-KD_MAVS-TM colocalized with the mitochondrial staining dye MitoTracker, indicating that the TM domain of MAVS targeted the Flag_PERK-KD_MAVS-TM protein to the mitochondria (Fig 2B). Following 90min treatment with rotenone, we observed a dose-dependent appearance of heterogeneous slower migrating bands of Flag_PERK-KD_MAVS-TM, which appeared most intense with 10 μM rotenone (Fig 2C, top panel). *In vitro* treatment with lambda phosphatase collapsed the heterogeneous slower-migrating bands of Flag_PERK-KD_MAVS-TM into a faster migrating band, confirming that the former represented autophosphorylated active PERK (Fig 2C, bottom panel). Time-course analysis revealed that Flag_PERK-KD_MAVS-TM phosphorylation was readily detected 30min after rotenone treatment and similar observations were made following treatment with antimycin A (Fig 2D); an inhibitor of the mitochondrial respiratory chain complex III that also increased mROS levels (S1 Fig) [33].

The isolated PERK-KD is enzymatically-active *in vitro* [32], raising the possibility that a diffusible signal(s) generated in rotenone treated cells might directly contribute to activation of the PERK kinase domain (independently of membrane anchoring via the TM domain). To test this possibility, we expressed FV2E-PERK, a previously characterized cytosolic fusion protein between PERK-KD and a modified FK506 binding domain (Fv2E) that undergoes ligand-induced activation [27]. Treatment with the dimerizing ligand AP20187 caused Fv2E-PERK phosphorylation, reflected in a shift in its mobility (Fig 2E). However, the cytosolic Fv2E-PERK was unresponsive to rotenone and antimycin A treatment. As FV2E-PERK lacks a TM domain and is cytosolic, these results demonstrate that Flag_PERK-KD_MAVS-TM phosphorylation requires membrane tethering via a TM domain and is likely mediated by mROS-induced changes in the outer mitochondrial membrane lipid bilayer properties and not by a diffusible signal. As PERK-KD autophosphorylation requires oligomerization, these results indicate that mROS inducers cause lipid-dependent MAVS-TM oligomerization [34]. In agreement with a role of ROS in triggering lipid-dependent MAVS-TM oligomerization, treatment with hydrogen peroxide (H_2_O_2_) also led to Flag_PERK-KD_MAVS-TM phosphorylation (Fig 2F).

**Fig 3:**
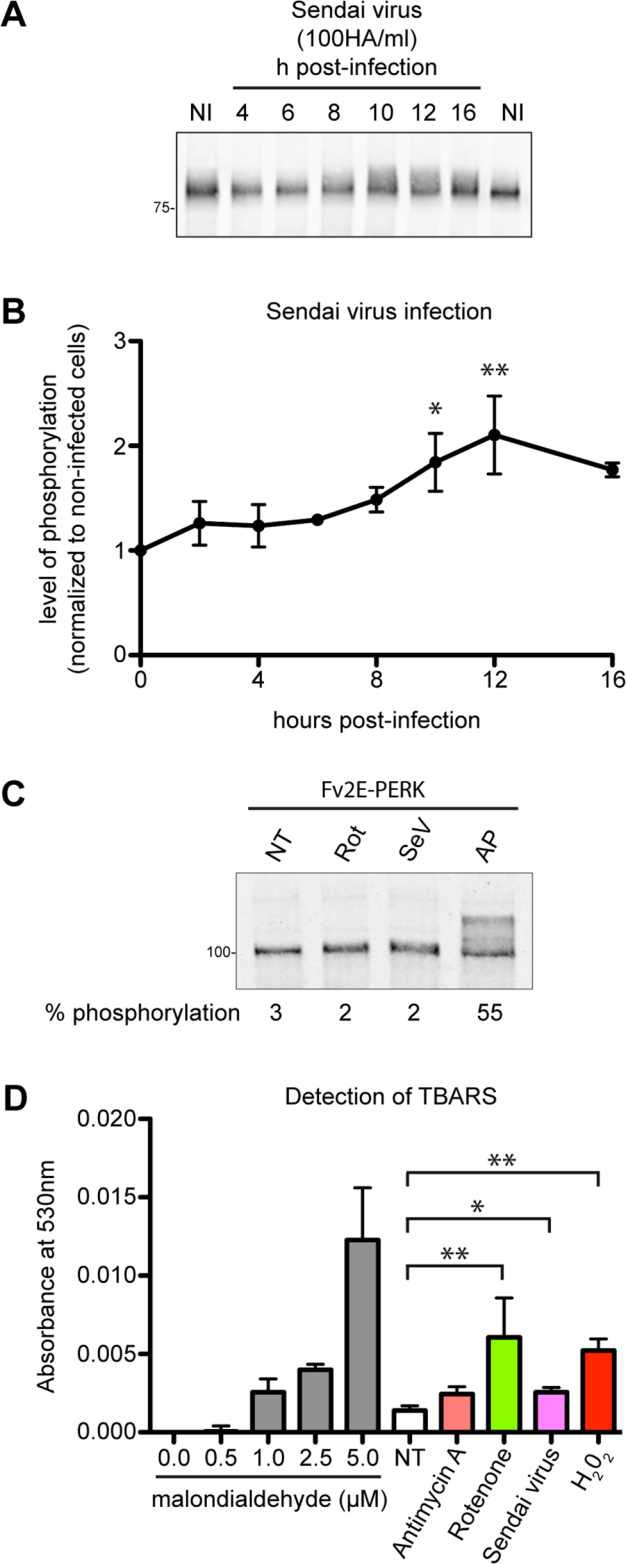
Sendai virus infection promotes lipid-dependent MAVS-TM domain oligomerization and increases lipid peroxidation. **(A)** Immunoblot of Flag_PERK-KD_MAVS-TM immunopurified from Flp-In T-Rex-293 cells infected with 100 HA/ml of Sendai virus strain Cantell for the indicated time. **(B)** Quantification of the level of Flag_PERK-KD_MAVS-TM phosphorylation in Sendai virus-infected cells. Shown are mean ± sem values of three independent experiments. *, p ≤ 0.05; **, p ≤ 0.01 **(C)** Immunoblot of Fv2E-PERK immunopurified from cells treated with 10 μM rotenone (Rot) for 90 min, or infected with 100 HA/ml Sendai virus for 16h (SeV), or treated with the Fv2E-PERK dimerizing ligand AP20187 (AP) at 10 nM for 90 min. The percentage of Fv2E-PERK phosphorylation is indicated under each sample. **(D)** Detection of lipid peroxidation end products by the Thiobarbituric Acid Reactive Substances (TBARS) assay. Pure malondialdehyde was used as a reference in the assay. TBARS were quantified from Flp-In T-Rex-293 cells treated for 90min with 40 μg/ml Antimycin A, 10 μM Rotenone, 1 mM hydrogen peroxide, or infected with 100 HA/ml Sendai virus for 16 hours. Shown is a representative experiment reproduced three times. Data are mean ± sem values of two technical replicates, each analysed in triplicate. *, p ≤ 0.05; **, p ≤ 0.01

Increased mROS production has been observed in virus-infected cells [35–37], prompting us to analyse whether ROS-mediated lipid membrane modifications correlate with Flag_PERK-KD_MAVS-TM activation in virus-infected cells. Increased Flag_PERK-KD_MAVS-TM phosphorylation was noted in cells infected with Sendai virus strain Cantell (Fig 3A) that was associated with increased mROS production, as shown by MitoSOX staining (S1 Fig). As FLAG_PERK-KD_MAVS-TM lacks the cytosolic domain of MAVS, it does not bind RIG-I and MDA-5 and therefore reports on lipid-mediated MAVS TM domain-dependent oligomerization in infected cells. The cytosolic fusion protein FV2E-PERK was not activated by Sendai virus infection, demonstrating that TM domain-mediated membrane tethering is required for Sendai virus-induced FLAG_PERK-KD_MAVS-TM activation (Fig 3C).

Sendai virus infection was associated with increased lipid peroxidation detected by the Thiobarbituric Acid Reactive Substances (TBARS) assay (Fig 3D) [38]. TBARS were also elevated in antimycin A, rotenone and H_2_O_2_-treated cells confirming that lipid peroxidation occurs under these conditions. While we cannot exclude a role for other mitochondrial membrane modifications that might contribute to MAVS-TM oligomerization in Sendai virus infected cells, or in cells treated with the mROS inducers rotenone and antimycin A, these observations are consistent with a role for lipid peroxidation in stimulating MAVS-TM oligomerization.

**Fig 4:**
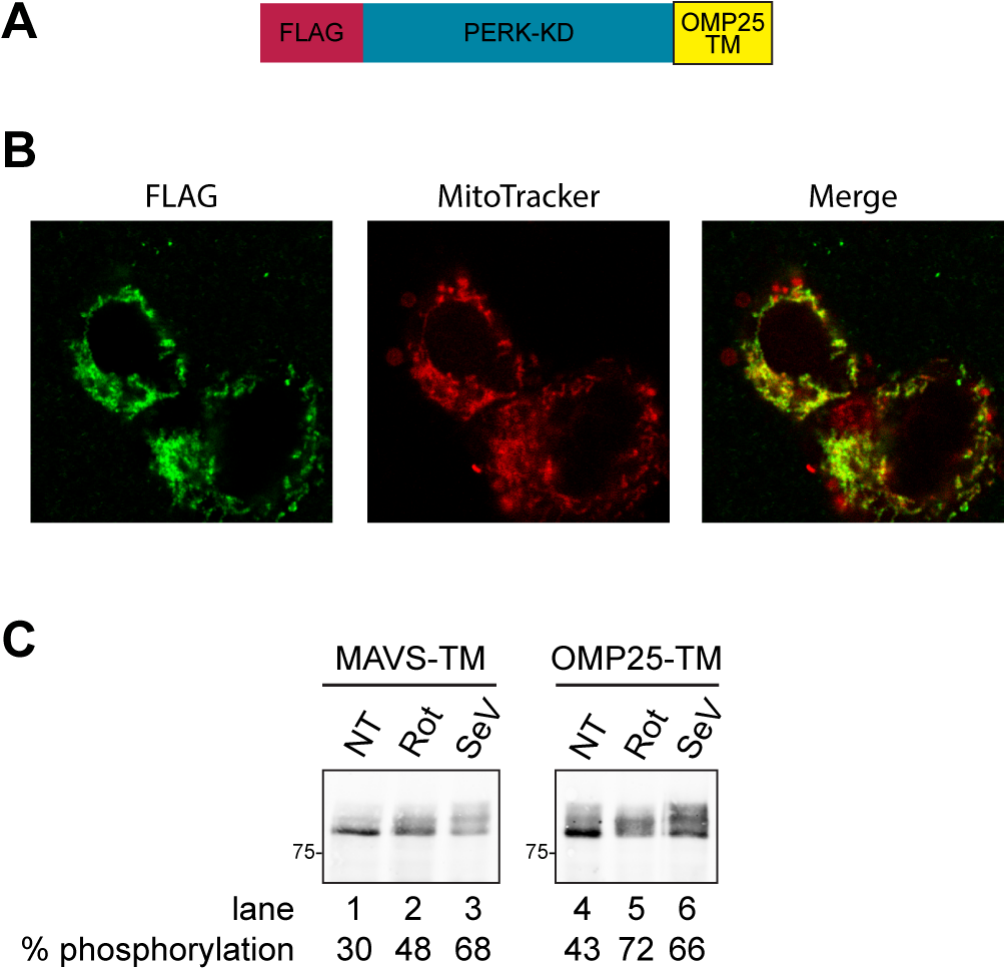
Rotenone and Sendai virus infection trigger oligomerization of the TM domain of the unrelated outer mitochondrial membrane protein OMP25. **(A)** Schema of Flag_PERK-KD_OMP25-TM, a N-terminally Flag-tagged fusion protein between the kinase domain of PERK and the TM domain of OMP25, an outer mitochondrial membrane protein. The construct encoding this protein was introduced into Flp-In T-Rex-293 cells by recombination **(B)** Intracellular localization of Flag_PERK-KD_OMP25-TM following doxycycline induction in Flp-In T-Rex-293 cells. Cells were stained for the Flag tag and the mitochondrial specific dye MitoTracker. Images were acquired sequentially on a confocal microscope. The merge panel shows an overlap of Flag and Mitotracker signals. **(C)** Immunoblot of Flag_PERK-KD fused to the TM domain of MAVS (MAVS-TM) or to the TM domain of OMP25 (OMP25-TM). Cells were treated with 10 μM rotenone (Rot) for 90 min, or infected with 100 HA/ml Sendai virus for 16h (SeV). The percentage of PERK-KD phosphorylation is indicated under each sample.

To determine if rotenone and Sendai virus-induced TM oligomerization is restricted to the TM domain of MAVS, we fused Flag_PERK-KD to the TM domain of OMP25, an unrelated tail-anchored outer mitochondrial membrane resident protein (Fig 4A). We introduced this construct by recombination in the Flp-In T-Rex-293 cell line and stimulated the expression of the fusion protein by doxycycline induction. The fusion protein between Flag_PERK-KD and OMP25 TM domain colocalized with the mitochondrial marker Mitotracker, confirming that the OMP25 TM domain is able to target a fusion protein to the mitochondria [4] (Fig 4B). We observed increased phosphorylation of PERK-KD fused to OMP25 TM domain in cells treated with rotenone and in cells infected with Sendai virus, reflecting increased lipid-dependent oligomerization mediated by the OMP25 TM domain under these conditions (Fig 4C). Thus, lipid-dependent TM domain oligomerization is not restricted to MAVS-TM domain, suggesting that rotenone treatment and Sendai virus infection induced changes in the OMM properties could modulate oligomerization of other mitochondrial proteins.

## Discussion

MAVS-TM domain is shown here to oligomerize in response to changes in the outer mitochondrial lipid membrane properties caused by treatment with mROS inducers or by Sendai virus infection. As MAVS oligomerization regulates its function, these results call attention to the potential role of the outer mitochondrial lipid membrane properties as a modulator of MAVS-mediated antiviral innate immune signalling.

**Fig 5:**
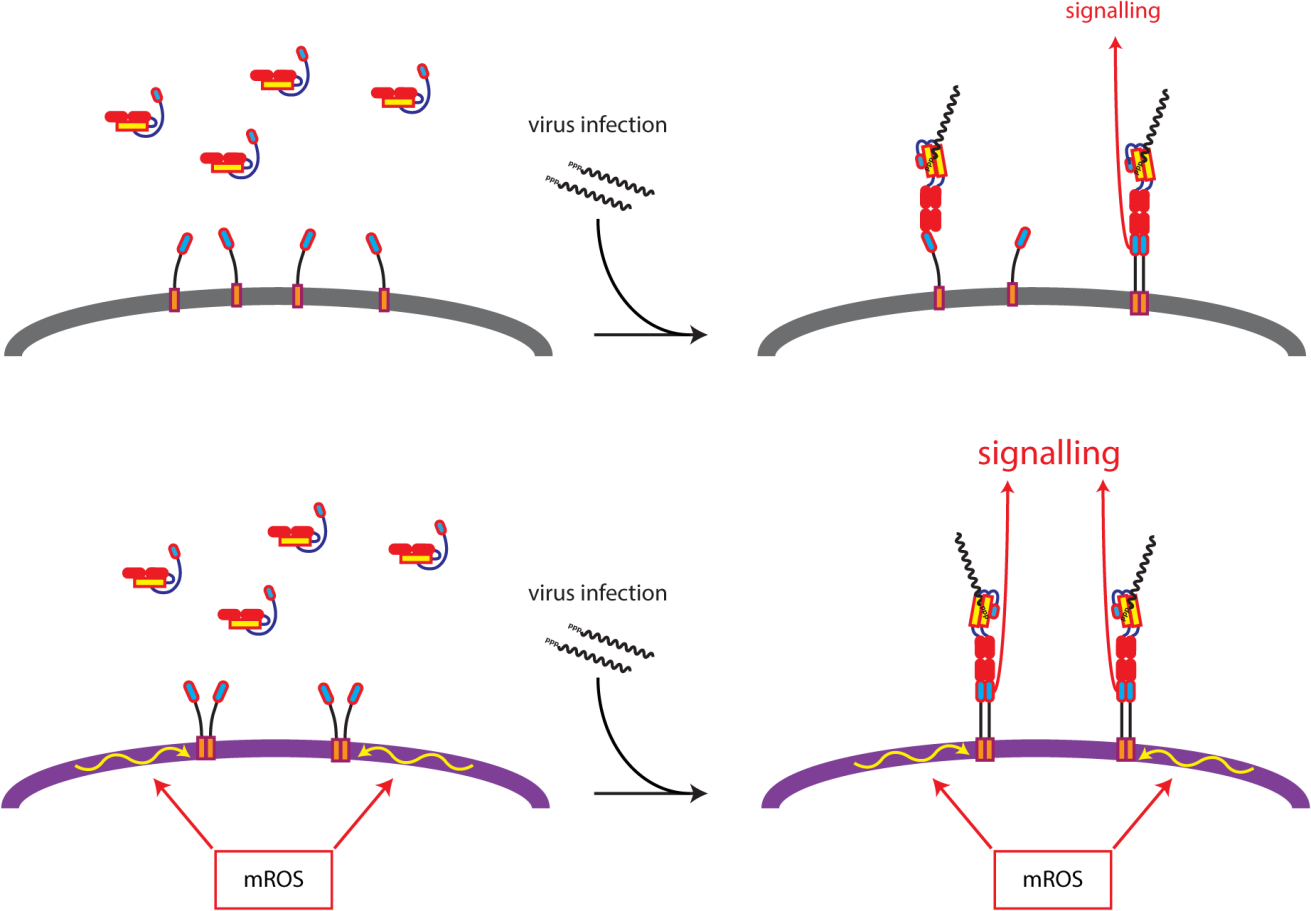
Proposed model for mROS effects on MAVS signalling. MAVS is predominantly monomeric on the outer mitochondrial membrane of cells with low levels of mROS (top panel). Increased mROS production promotes lipid-dependent oligomerization of MAVS TM domain (lower panel). Upon viral infection, RLRs bind to viral RNA and expose their CARD domain, which is now available to interact with the CARD domain of MAVS. RLR induced MAVS CARD domains homo-oligomerization is required for MAVS signalling. Lipid-dependent TM domain oligomerization could prime MAVS for RLR-induced MAVS CARD domains homooligomerization, therefore leading to increased MAVS signalling.

The mROS inducer rotenone potentiated IFN-β and CXCL10 mRNA induction in response to concomitant application of low concentrations of the RLR agonist poly(I:C)-LV. Interestingly, when applied alone rotenone was insufficient to activate type I IFNs transcription but was able to activate an oligomerization reporter construct (Flag_PERK-KD_MAVS-TM) localised to the outer mitochondrial membrane. Both events are dependent on oligomerization of their respective effector: Oligomerization of the cytosolic CARD domain of MAVS promotes downstream signalling to the IFNs genes, whereas oligomerization of PERK kinase domain is required for autophosphorylation in the chimeric reporter. The differences between the responses of the two are consistent with different thresholds of sensitivity to the oligomer-monomer equilibrium, with the MAVS requiring sustained oligomerization reinforced by concomitant RLR binding to trigger downstream signalling. Thus mROS inducers favour MAVS-TM oligomerization but at a level that is insufficient to cause the sustained MAVS CARD domain interaction needed for downstream signalling [39]. Nonetheless, our findings suggest that mROS-induced lipid-dependent MAVS TM domain oligomerization could lower the threshold of RLR ligand required for MAVS CARD homodimerization or homooligomerization and therefore prime MAVS for signalling (Fig 5).

The endoplasmic reticulum lipid membrane properties have been shown to modulate signalling by the unfolded protein response (UPR) transducers, IRE1 and PERK [24,40,41]. Lipid modifications triggered oligomerization of IRE1 and PERK TM domains, as observed here for MAVS-TM domain, indicating that lipid-dependent TM domain oligomerization may be a conserved mechanism modulating TM protein function. Changes in the lipid membrane properties causing TM domain-dependent oligomerization synergize with concomitant lumenal (unfolded protein) or cytosolic (RLR) oligomerizing signals to promote downstream signalling to effectors. However, the UPR transducers have a lower threshold of sensitivity to the oligomer-monomer equilibrium than MAVS, with PERK and IRE1 being able to respond to changes in the endoplasmic reticulum lipid composition independently of any concomitant lumenal unfolded protein signal [24].

Oligomerization was observed for the TM domain of MAVS and OMP25, which are two unrelated TM peptides that have a similar hydrophobicity profile, but that to our knowledge do not share any noticeable common peptide motif. As the amino acids constituting the TM domain seemed to have a minor role in conferring responsiveness to rotenone or Sendai virus induced lipid modifications, oligomerization is not likely to rely on specific peptide-lipid interactions, bur rather relies primarily on changes in the biophysical properties of the lipid membrane. Changes in the lipid bilayer biophysical properties known to modulate TM domain oligomerization include modifications of the thickness of the lipid bilayer, changes in membrane fluidity and acyl chain flexibility. These lipid bilayer biophysical properties affect the strength of the interaction between TM amino acid side chains and the surrounding lipids. When the strength of the peptide-lipid interaction decreases, polar amino acids embedded in the lipid bilayer are no longer solvated as efficiently by surrounding lipids, creating a thermodynamically unfavourable situation that can be relieved by the interaction between amino acid side chains from TM peptides [42]. This generic mechanism can promote TM-TM interactions in response to changes in the biophysical properties of the membrane. At present, the nature of the changes in the OMM biophysical properties occurring in cells treated with mROS inducers or in Sendai virus infected cells remain to be characterized.

The mitochondrial phospholipid composition has been extensively studied and reviewed [43–45]. In addition to the most prevalent phospholipids phosphatidylcholine and phosphatidylethanolamine, mitochondria contain the mitochondria specific phospholipid cardiolipin, which accounts for around 15–20% of total mitochondrial phospholipids. Cardiolipins accumulate in the inner mitochondrial membrane (IMM). The proportion of cardiolipin in the OMM ranges from trace amounts to around 25% of total cardiolipins depending on the studies [43,46]. This variability is in part due to the technically challenging differential purification of IMM and OMM. The mitochondrial lipid membrane properties are also determined by the acyl chain length and degree of unsaturation within each phospholipid class. Cardiolipins have a high degree of acyl-chain unsaturation, rendering them highly susceptible to ROS-mediated oxidation. The acyl chain composition of the other mitochondrial phospholipids remains poorly defined. When the phospholipid class is not considered, total mitochondrial phospholipids have around 14% of mono unsaturated acyl chains and around 49% of polyunsaturated acyl chains irrespective of the phospholipid class [43]. The relative proportions in the IMM and OMM are not known, but it is thought that unequal repartition of phospholipid classes and acyl chains between the IMM and the OMM are important for maintaining a smooth OMM and a folded less fluid IMM [45]. Even within the mitochondrial membranes, lipid distribution is heterogeneous, as observed in the cardiolipin rich cristae tips or in the mitochondrial-associated membranes microdomains [44], which are contact sites between the ER and the OMM recently involved in antiviral signalling [5]. Alterations in the lipid composition can impact mitochondrial membrane protein function and the regulation of mitochondrial morphology and dynamics. Treatment with rotenone has been associated with the oxidation of mitochondrial lipids [47], but also with alterations in mitochondrial lipids distribution [48] and with changes in the mitochondrial morphology [49]. All these changes could potentially occur in mROS inducers-treated cells or in Sendai virus-infected cells and trigger changes to the biophysical properties of the OMM. Further work is required to determine which changes to the mitochondrial lipid properties cause MAVS-TM oligomerization.

Viruses rely on host cell metabolism for their replication. As a consequence, they are unlikely to develop resistance to or bypass the consequences of processes such as metabolism-induced ROS production that are linked to pathways needed for their replication. Thus, identifying ways to manipulate cellular metabolism to increase mROS production specifically in infected cells could be a promising approach to promote antiviral signalling. This could complement conventional PAMP detection mechanisms to strengthen the innate immune response by taking advantage of the specific metabolic requirements of a virus in a host cell.

## Supporting Information

S1 Fig Mitochondrial ROS production. Histogram of MitoSOX fluorescent signal from untreated Flp-In T-Rex-293 cells (blue curve) and cells treated for 90min with 10 μM rotenone (yellow curve), 40 μg/ml antimycin A (green curve), or infected with 16h with Sendai virus (orange curve). Cells were stained with MitoSOX and analysed by flow cytometry.
(TIF)
