# Supplementary figures and images for: Modulation of Innate Immune Signalling by Lipid-Mediated MAVS Transmembrane Domain Oligomerization
Source: PLoS One. 2015 Aug 28;10(8):e0136883. doi: 10.1371/journal.pone.0136883 (PMC4552940; doi:10.1371/journal.pone.0136883)

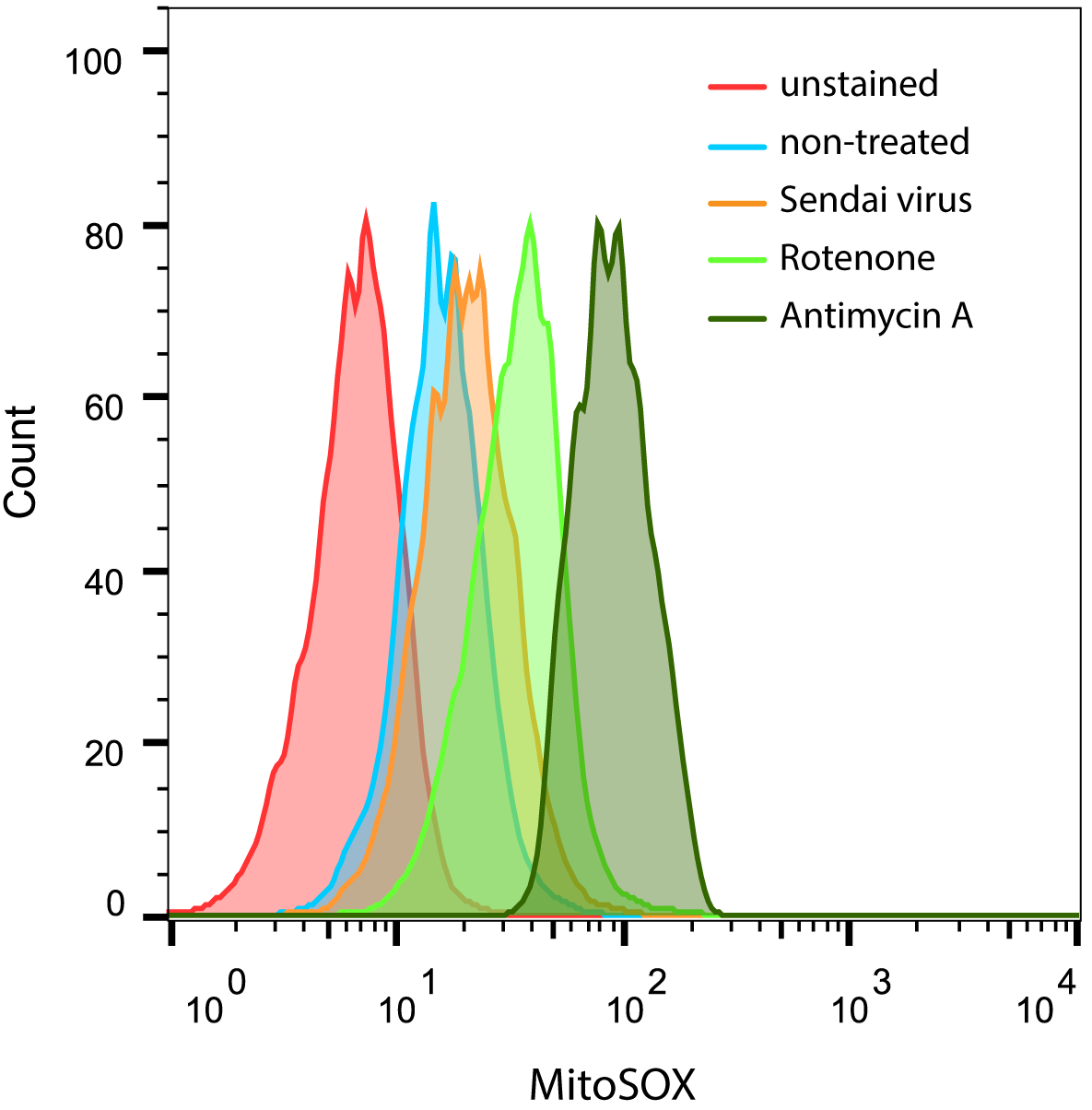

Supplement: S1 Fig — Histogram of MitoSOX fluorescent signal from untreated Flp-In T-Rex-293 cells (blue curve) and cells treated for 90min with 10 μM rotenone (yellow curve), 40 μg/ml antimycin A (green curve), or infected with 16h with Sendai virus (orange curve). Cells were stained with MitoSOX and analysed by flow cytometry. (TIF) [file pone.0136883.s001.tif]
